# Isolated Oral Mucosal Zoster With Facial Palsy: A Case Report

**DOI:** 10.7759/cureus.33472

**Published:** 2023-01-06

**Authors:** Hüsna Güder, Aziz A Hamidi, Nilgun Cinar

**Affiliations:** 1 Dermatology, Maltepe University, Istanbul, TUR; 2 Infectious Disease, Fatih Sultan Mehmet Training and Research Hospital, Istanbul, TUR; 3 Neurology, Maltepe University, Istanbul, TUR

**Keywords:** oral ulcer, acute pain, facial palsy, oral diseases, herpes zoster virus

## Abstract

Herpes zoster (HZ) is an infection characterized by the appearance of unilateral painful vesicular lesions on the skin and mucous membranes. Facial paralysis is one of the complications of HZ. The diagnosis of HZ can be easily missed when there is no lesion on the skin. We present a rare case with isolated oral mucosal lesions accompanied by facial palsy.

## Introduction

Unilateral painful vesicular lesions appear on the skin and mucosae in herpes zoster (HZ) due to varicella-zoster virus (VZV) reactivation. Herpes zoster's area of involvement is most commonly thoracic, lumbar, cervical, and sacral, respectively. Oral mucosa involvement is rare. Herpes zoster may have many complications such as secondary bacterial infections, postherpetic neuralgia, scarring of the skin, keratitis, retinal necrosis, cranial and peripheral nerve palsies, cerebral ataxia, and pneumonia. Early diagnosis and treatment are crucial in preventing and reducing the severity of complications [1]. 

## Case presentation

**Figure 1:**
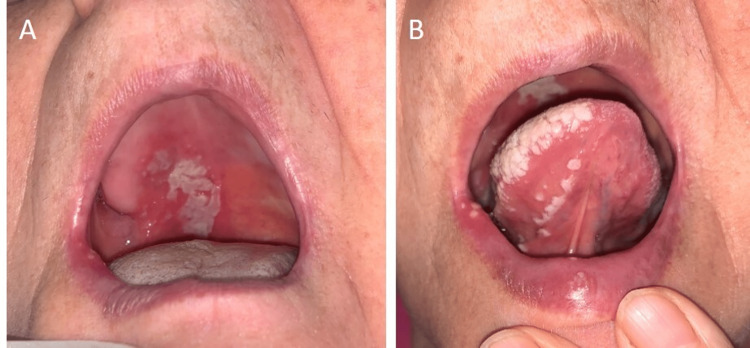
Lesions of the patient at the time of admission Unilateral erythematous ulcers and white plaque in her palate (A) and tongue (B)

**Figure 2:**
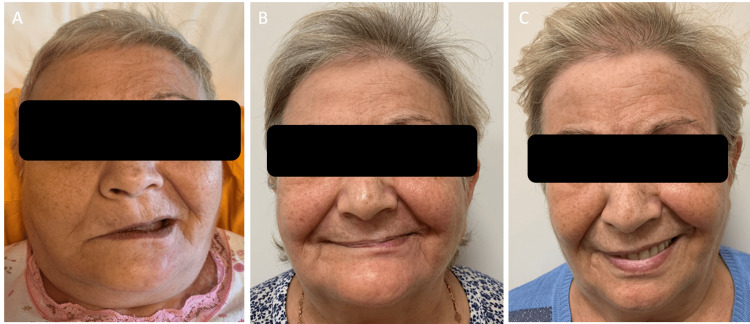
The appearance of ipsilateral facial paralysis in the patient A: During admission, B: Three months later, C: Two years later

A 57-year-old female patient presented with sores in the mouth for five days and severe pain on one side of the head. The patient had undergone thyroidectomy six years ago and is using levothyroxine sodium. She was healthy otherwise. Unilateral erythematous ulcers on her palate and tongue in addition to white plaque were observed on dermatologic examination, and she had ipsilateral peripheral facial paralysis (Figure 1 A & B, and Figure 2 A). The stage of the patient's facial paralysis was consistent with stage 5 according to the House Brackmann staging [2]. She had anosmia and tinnitus for three days and hypoesthesia on the side of the paralysis on her face. Sedimentation was high (40 mm/h). Blood biochemistry is normal. Anti-HIV antibody and Covid-19 polymerase chain reaction (PCR) test were negative. A cerebral magnetic resonance imaging scan was performed and showed normal results. We started intravenous acyclovir treatment, and on the second day of the treatment, methylprednisolone was added for facial paralysis. We started methylprednisolone at a daily dose of 64 mg and ended it with a gradual dose reduction within one month. Non-steroidal anti-inflammatory agents and vitamin B12 were added. The patient's lesions and pain decreased within five days of treatment, but facial paralysis did not resolve completely. Electromyography was done after the first month of the treatment. Complete paresis was detected in the frontalis and orbicularis oculi muscles. In addition, partial paresis was seen in the orbicularis oris muscles, and a physiotherapy program was performed. Three months later, the patient's facial palsy stage regressed to stage 4, and anosmia and tinnitus resolved (Figure 2 B). Two years later, the patient's facial palsy stage regressed to stage 3 (Figure 2 C).

## Discussion

The incidence of HZ increases with age. In one study, the incidence of HZ was found to be 7.20 per 1000 persons in the 50 to 54 years age group, while this value was found to be 13.99 per 1000 person-years in the >80 age group. Among patients with HZ, the rate of neurological complications was reported as 0.77% to 1.36%. Common neurological complications include Bell-like palsy/motor nerve palsy, sensory loss, and cranial nerve palsy [3]. Our patient was 57 years old and accompanied by facial paralysis.

There are few reports of oral involvement in HZ [4-7]. Our case is the first describing HZ with isolated oral lesions and accompanying facial paralysis.

In a study examining 142 patients with acute peripheral facial paralysis, it was reported that typical zoster lesions in the ear or mouth epithelium were present in 13 of the patients, while the lesions appeared later in eight patients [8]. Varicella zoster reactivation was detected with PCR analysis in 35 of the remaining 121 patients without zoster lesions. It was emphasized that VZV is one of the main etiological agents of Bell's palsy [8]. In another study, VZV PCR was studied from the saliva of 171 patients with Bell's palsy, and a 1.7% positivity rate was found [9]. The fact that different results were obtained in these publications examining the relationship between HZ and facial paralysis indicates that more studies are needed on this subject.

In patients with facial paralysis, the diagnosis of HZ can be missed if there are no obvious lesions on the outer ear canal or oral mucosa when a careful examination is not performed.

## Conclusions

Isolated zoster in the oral mucosa is rare, which may lead to diagnosis delays. Considering zoster in the presence of unilateral painful oral mucosal lesions is important for early initiation of treatment and prevention of complications.
